# Reevaluating Auditory Hallucinations: Beyond the Psychotic Disorder Paradigm

**DOI:** 10.1192/j.eurpsy.2025.748

**Published:** 2025-08-26

**Authors:** V. Alves Barata, J. Bastos, J. Revez Lopes, M. Palma

**Affiliations:** 1 Hospital Prof. Doutor Fernando Fonseca; 2 Hospital de Santa Maria, Lisbon, Portugal

## Abstract

**Introduction:**

Auditory hallucinations (AH) are frequently considered a hallmark of psychotic disorders. Even in the absence of any other features, persistent AH will fall under the DSM-5 label of Other Specified Schizophrenia Spectrum and Other Psychotic Disorder. However, AH are not exclusive to psychosis and can occur across various psychiatric and neurological conditions. Furthermore, AH have been reported in the general population, with prevalence estimates ranging from 4% to 21%. While typically transient and sporadic, a minority may experience recurrent and persistent AH. These experiences, spanning from subclinical to pathological, have been lately understood within the framework of the “extended psychosis phenotype”.

**Objectives:**

This study aims to challenge the conventional view of AH as definitive indicators of psychotic disorders by examining their occurrence in different contexts and exploring the relevance of the “extended psychosis phenotype” in understanding these symptoms.

**Methods:**

A literature review was conducted using the keywords “auditory hallucinations”, “extended psychosis phenotype” and “phenomenology” in the PubMed and Google Scholar databases.

**Results:**

Psychotic experiences seem to run in families, suggesting a transdiagnostic psychosis trait that may be passed down independently of emotional or thought regulation processes. As a result, psychosis - and AH in particular - can be expressed across multiple disorders, including schizophrenia, bipolar disorder, major depressive disorder, anxiety disorders, autism spectrum disorders, post-traumatic stress disorder (PTSD), and certain personality disorders, such as borderline personality disorder (BPD). Phenomenological differences of AH across these conditions remain unclear. Notably, evidence challenges the concept of ‘pseudohallucinations’ in BPD, showing that AH can be as severe and persistent as those in schizophrenia. Neurobiologically, AH are not always linked to abnormal dopamine activity, which calls into question the routine use of antipsychotics for all psychotic-like symptoms. Environmental and psychological factors, such as trauma, also play a role in AH, especially in BPD and PTSD. In such cases, psychosocial interventions, such as cognitive-behavioral therapy and trauma-focused therapies, are often more effective than pharmacological treatments. Finally, AH can occur in isolation, with a generally low risk of progressing into a full psychotic disorder unless accompanied by other psychotic symptoms or functional impairments.

**Conclusions:**

The diagnostic approach to AH should be reconsidered to avoid automatic narrowing of differential diagnosis to psychotic disorders. AH can emerge from various mechanisms, including non-dopaminergic pathways. Recognizing the extended psychosis phenotype and transdiagnostic psychosis trait is crucial for understanding the continuum of psychotic experiences and improving treatment approaches.

**Disclosure of Interest:**

None Declared

